# Simulation Study on the Isothermal Aging Precipitation Process of Al_3_Sc in Al-Sc Alloys Using a High-Resolution Population Dynamics Model

**DOI:** 10.3390/ma19102175

**Published:** 2026-05-21

**Authors:** Hao Xiong, Yufei Zhao, Wenyi Hao, Zhenzhi Sun, Xuechun Wang, Yao Xiao, Pengliang Ji, Guodong Fan

**Affiliations:** 1 National Engineering Research Center for Equipment and Technology of Cold Strip Rolling, Yanshan University, Qinhuangdao 066004, China; 2 School of Mechanical Engineering, Yanshan University, Qinhuangdao 066004, China; 3 Hebei Yanzhao Lantian Steel Sheet Group Co., Ltd., Hengshui 053500, China; 4 School of Mechanical Engineering, University of Science and Technology Beijing, Beijing 100083, China; 5 Shougang Group Co., Ltd., Beijing 100043, China

**Keywords:** precipitation, high-resolution algorithm, population dynamics model, Al-Sc alloy, volume fraction

## Abstract

Al-Sc alloys are widely applied in aerospace and automotive lightweighting owing to the excellent performance imparted by nano-sized Al_3_Sc precipitates. Accurate simulation of the full-cycle precipitation kinetics is critical for optimizing aging heat treatment processes, but the traditional Lifshitz-Slyozov-Wagner (LSW) theory is only applicable to the coarsening stage, while the conventional Kampmann-Wagner-Numerical (KWN) model suffers from severe numerical diffusion and fails to correct errors caused by discontinuous precipitate size distributions. To address these issues, a high-resolution population dynamics model based on the Van Leer limiter was established in this study, which is an improved KWN model that simultaneously considers interfacial energy transition during nucleation and coarsening and the effect of precipitate volume fraction on particle growth rate. Isothermal aging precipitation of Al_3_Sc in Al-0.2 wt.% Sc and Al-0.3 wt.% Sc alloys at 350 °C was systematically simulated, and key kinetic parameters including nucleation rate, critical nucleation radius, average precipitate radius, and normalized size distribution were calculated. The results show that the simulated average radius and normalized size distribution are in excellent agreement with experimental data, and the model accurately captures the plateau characteristic of average radius evolution during aging. Increasing Sc content significantly shortens the nucleation-growth stage and advances the onset of coarsening by approximately one order of magnitude. Compared with the LSW theory, the proposed model achieves second-order accuracy in smooth regions and suppresses spurious oscillations in discontinuous regions, fully reproducing the incubation, nucleation-growth, and coarsening stages of precipitation. This high-resolution model provides reliable theoretical support for the aging process optimization of Al-Sc alloys and offers an effective numerical method for precipitation kinetics simulation of other dilute binary alloys.

## 1. Introduction

As a high-performance lightweight structural material, Al-Sc alloys exhibit broad application prospects in aerospace, rail transit, automotive lightweighting and other fields owing to the excellent grain refinement effect, room-temperature strength, high-temperature creep resistance and recrystallization stability imparted by nanoscale Al_3_Sc precipitates [1,2,3,4]. The mechanical performance of aluminum-scandium alloys is directly determined by the evolutionary changes in the dimensions, number concentration, particle size distribution, and microstructure morphology of Al_3_Sc precipitates throughout the aging heat treatment process. A core prerequisite for the precise control of aging processes is to establish a complete and accurate quantitative description of the precipitation kinetics of the Al_3_Sc phase [5,6,7,8].

Currently, research on the aging precipitation kinetics of alloys is mainly divided into two categories: experimental characterization and numerical simulation [9]. Experimental characterization enables the acquisition of precipitate characteristic parameters through techniques such as transmission electron microscopy (TEM) and three-dimensional atom probe (3D-AP). Nevertheless, it is difficult to track the dynamic evolution of the three consecutive stages (nucleation, growth, and coarsening) across the full aging cycle in real time. Moreover, experimental approaches entail high costs and long timeframes [10]. Numerical simulation enables quantitative prediction of the full-process precipitation kinetics and provides efficient theoretical guidance for the optimization of aging processes, which has made it a core research approach in this field [11,12,13].

The classical Lifshitz-Slyozov-Wagner (LSW) theory is the earliest theoretical model used to describe the coarsening behavior of precipitates. However, it is only applicable to the coarsening stage in infinitely dilute systems where the precipitate volume fraction approaches zero, and cannot cover the nucleation and growth processes in the early aging period. Furthermore, it fails to effectively predict the plateau regime in the evolution of the average precipitate radius, which is commonly present in dilute Al-Sc alloys [14]. To address this issue, the Kampmann-Wagner-Numerical (KWN) model has been proposed and widely adopted [15,16,17]. This model couples the three stages of nucleation, growth and coarsening without introducing simplifying assumptions for the precipitation process, and enables simulation of precipitation kinetics throughout the entire aging cycle. It has been applied to investigate the precipitation behavior in various aluminum alloy systems [18,19]. However, the conventional KWN model takes particle number density as the solution variable, and its governing equations are incompatible with high-order high-resolution numerical algorithms. Conventionally, this model is solved with the first-order upwind scheme, which readily triggers serious numerical diffusion. It is difficult to correct the computational deviations arising from the discontinuity in the precipitate size distribution during the nucleation stage, leading to significant discrepancies between the simulation results and experimental data.

To address the shortcomings of the conventional KWN model, the population dynamics model based on the size distribution function, as an improved KWN model, has attracted extensive attention in the academic community [20,21,22,23]. This model adopts the size distribution function (in the form of an intensive property) as the solution variable, and its governing equation is a hyperbolic equation with a source term. It is compatible with high-order numerical algorithms and holds the potential to enhance computational accuracy. However, most existing precipitation simulation studies do not simultaneously account for the interfacial energy transition during nucleation and coarsening, nor the inter particle competitive growth effect caused by the precipitate volume fraction [24]. Furthermore, no high-resolution solution schemes that balance computational accuracy and stability have been developed to address the spurious oscillations and numerical diffusion caused by the discontinuity in the size distribution during the nucleation stage. Consequently, the model fails to accurately reproduce the full cycle precipitation kinetic characteristics of Al-Sc alloys throughout the aging process.

Accordingly, this study establishes a high-resolution population dynamics model based on the Van Leer limiter, which simultaneously incorporates the interfacial energy transition during the nucleation-coarsening stage and the influence of precipitate volume fraction on particle growth rate. Systematic simulations are conducted on the precipitation behavior of the Al_3_Sc phase in Al-0.2 wt.% Sc and Al-0.3 wt.% Sc alloys under isothermal aging at 350 °C. Key kinetic parameters of precipitates during aging, including nucleation rate, critical nucleation radius, average radius, and normalized size distribution, are calculated. The simulation results are compared and validated against published experimental data and computational results from the conventional LSW theory, and the regulatory effect of Sc content on the aging precipitation kinetics of Al-Sc alloys is clarified. This study provides a high-precision theoretical prediction tool for the optimization of aging heat treatment processes for Al-Sc alloys, and also offers a reference for the precipitation kinetic simulation of other dilute binary alloy systems.

## 2. Methodology

Simulating the precipitation behavior of precipitated phases during alloy aging is essential to model the precipitation process of precipitates in supersaturated solid solutions. Most precipitated phases are metallic compounds, some of which are formed by the clustering of solute atoms. Their evolution involves three stages: nucleation, growth and coarsening. These three stages do not occur independently but take place concomitantly. Simulation model systems based on such a three-stage coupled mechanism have been extensively investigated, among which the KWN model and its improved variants have garnered widespread attention.

### 2.1. KWN Model

The KWN model discretizes the radii of precipitate particles into multiple size intervals and partitions the aging time into several independent time steps. If the variation in variables such as supersaturation is sufficiently small within a single time step, the nucleation rate and growth rate can be treated as constant values, thereby enabling the calculation of the particle count in each size interval. Let $N_{i}\left(t\right)$ represent the particle count per unit volume within the specified size range $\left[R-∆R/2,R+∆R/2\right]$ at time *t*. The particle size distribution is discretized into separate intervals according to particle radii to calculate the value of $N_{i}\left(t\right)$. The governing equation of the KWN model is as follows [25,26]:

(1) $$\frac{\partial N_{i}\left(t\right)}{\partial t}+\frac{\partial \left[v\left(R,t\right)N_{i}\left(t\right)\right]}{\partial R}=I\left(t\right)$$

where $v\left(R,t\right)$ is the growth rate of precipitate particles at time *t* with radius *R* (m/s), $N_{i}\left(t\right)$ is the number of precipitate particles in the *i*-th size interval per unit volume at time, and $I\left(t\right)$ is the nucleation rate at time t.

The first-order upwind scheme is employed to compute the evolution of the precipitate count within the *i*-th size interval, based on the number density and growth rate of neighboring size categories. The corresponding computational formula is presented as follows:

(2) $$\begin{array}{c} N_{i}\left(t+∆t\right)=N_{i}\left(t\right)+\frac{∆t}{∆R}v_{i-\frac{1}{2}}\left(t\right)\{sgn[v_{i-\frac{1}{2}}\left(t\right)]N_{i-1}\left(t\right)+sgn[-v_{i-\frac{1}{2}}\left(t\right)]N_{i}\left(t\right)\}\\ -\frac{∆t}{∆R}v_{i+\frac{1}{2}}\left(t\right)\{sgn[v_{i+\frac{1}{2}}\left(t\right)]N_{i}\left(t\right)+sgn[-v_{i+\frac{1}{2}}\left(t\right)]N_{i+1}\left(t\right)\} \end{array}$$

where $v_{i-\frac{1}{2}}\left(t\right)$ is the growth rate of precipitate particles at the left interface of the *i*-th size interval (m/s), $v_{i+\frac{1}{2}}\left(t\right)$ is the growth rate of precipitate particles at the right interface of the *i*-th size interval (m/s), and Δt is time step (s).

In Equation (2), when *x* > 0, $\mathrm{sgn}\left[x\right]\;=\;1$; when, $\mathrm{sgn}\left[x\right]\;=\;0$.

The KWN model achieves relatively high accuracy without introducing assumptions or approximations in the calculation process. It can be used to determine key precipitation parameters of alloys and to verify the applicability of existing nucleation and growth theories to real alloys. However, since this model takes particle number as the calculation variable, its governing equation cannot be solved using high-resolution algorithms. Consequently, it is difficult to correct the computational deviations caused by the discontinuity of precipitate size distribution during the nucleation stage by improving computational accuracy.

### 2.2. Population Dynamics Model

Recently, an improved KWN model, namely the population dynamics model, has attracted considerable attention [20,21,22,23]. Compared with the conventional KWN model, this model does not directly solve for the particle number $N\left(R,t\right)$ within different size intervals, but instead solves for the size distribution function $f\left(R,t\right)$. The relationship between them is $N\left(R,t\right)=f\left(R,t\right)∆R$, and its governing equation is:

(3) $$\frac{\partial f(R,t)}{\partial t}+\frac{\partial [v\left(R,t\right)f\left(R,t\right)]}{\partial R}=\frac{\partial I(t)}{\partial R}{|}_{R=R^{*}}$$

where $f\left(R,t\right)$ is the size distribution of precipitates at time *t* with radius *R*, and $R^{*}$ is the critical nucleation radius (m).

Equation (3) shows that the first term on the left-hand side stands for the time variation in the precipitate size distribution function, while the second term characterizes the influence of precipitate growth or dissolution on the distribution function $f\left(R,t\right)$. The right-hand side corresponds to the source term associated with precipitate nucleation.

As an improved variant of the KWN model, the population dynamics model also adopts no simplified assumptions or approximate treatments. The difference between the two models is that the size distribution function $f\left(R,t\right)$ is an intensive property, while the particle number $N\left(R,t\right)$ is an extensive property. Thus, the governing Equation (3) is a hyperbolic equation that includes a source term, where the right-hand side acts as the source term. This equation can be solved using various well-established high-order algorithms, such as the Lax-Wendroff scheme and other high-resolution schemes, which effectively eliminate the artificial diffusion generated by low-order numerical methods.

### 2.3. Aging Precipitation Process

#### 2.3.1. Nucleation

Nucleation is a multi-atom process in which atoms in the matrix aggregate to form critical clusters of stable precipitated phases. The fundamental driving force underlying nucleation arises from the variation in Gibbs free energy triggered by the formation of precipitated phases. This energy variation typically stems from the synergistic contribution of matrix interfacial energy and volume free energy. The algebraic expression for the total free energy change ΔG is as follows:

(4) $$∆G=\frac{4}{3}\pi R^{3}∆G_{v}+4\pi R^{2}\gamma$$

where $∆G_{v}$ is free energy for nucleation per unit volume (J/m^3^). It is explicitly noted that $∆G_{v}$ is a negative scalar, acting as the fundamental thermodynamic driving force for the precipitation process and γ is specific interfacial energy between precipitates and the matrix (J/m^2^).

**Figure 1:**
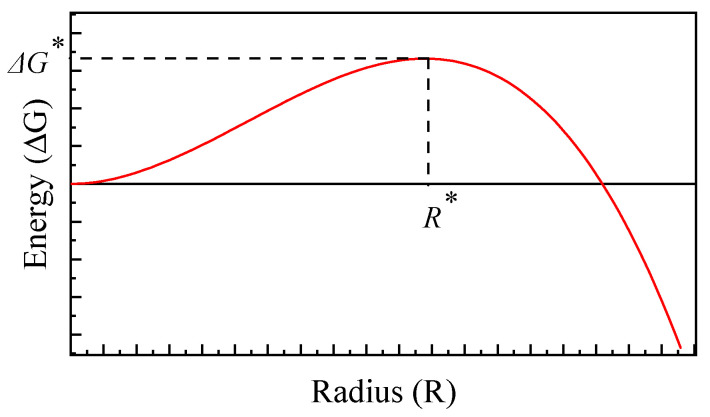
Schematic diagram of the relationship between free energy ∆G and radius R during nucleation of spherical precipitate particles.

Figure 1 shows a schematic diagram of the relationship between the free energy change ΔG and its radius R during the nucleation of spherical precipitate particles. Here, $\Delta G^{*}$ denotes the free energy change required to form the critical radius $R^{*}$. The critical free energy $\Delta G^{*}$ and critical radius $R^{*}$ are obtained at $\frac{\partial \left(\Delta G\right)}{\partial R}=0$. Differentiating Equation (4) yields:

(5) $$\frac{\partial (∆G)}{\partial R}=0=-4\pi R^{2}\phi +8\pi R\gamma$$

The critical nucleation radius $R^{*}$ and critical free energy $\Delta G^{*}$ can be expressed respectively as:

(6) $$R^{*}=\frac{-2\gamma }{∆G_{v}}$$

(7) $$∆G^{*}=\frac{16\pi \gamma^{3}}{3{∆G_{v}}^{2}}$$

By definition, the nucleation rate refers to the quantity of critical nuclei formed per unit time per unit volume of the untransformed matrix. Based on the classical nucleation theory for spherical precipitates, the correlation between the nucleation rate I and the isothermal reaction time t can be expressed as:

(8) $$I=\beta^{*}N_{V}Ze^{\left(\frac{-∆G^{*}}{K_{B}T}\right)}e\left(\frac{-\tau }{t}\right)$$

where $\beta^{*}$ is the impingement rate of atoms onto critical nuclei, $N_{V}$ is the density of nucleation sites in the matrix (number of atoms per unit volume), $K_{B}$ is the Boltzmann constant, T is the nucleation temperature (K), Z is the Zeldovich non-equilibrium factor, τ is the nucleation incubation time (s), and t is the isothermal reaction time (s).

The supplementary formula to Equation (8) is as follows:

(9) $$\beta^{*}=\frac{16\pi \gamma^{2}cD}{{\left(∆G_{v}\right)}^{2}a^{4}}$$

The Zeldovich non-equilibrium factor Z accounts for the probability of critical nuclei dissolving back into the matrix. It is fundamentally defined based on the second derivative of the free energy with respect to the number of atoms n at the critical nucleus size $n^{*}$, i.e., $Z=\sqrt{\frac{-1v_{\alpha }{\left(∆G_{v}\right)}^{2}}{2\pi K_{B}T}{\left(\frac{\partial^{2}∆G}{\partial n^{2}}\right)}_{n^{*}}}$. By substituting the critical parameters, it expands to the following analytical form:

(10) $$Z=\frac{v_{\alpha }{\left(∆G_{v}\right)}^{2}}{8\pi \sqrt{\gamma^{3}K_{B}T}}$$

(11) $$\tau =\frac{8K_{B}T\gamma a^{4}}{v_{\alpha }^{2}{\left(∆G_{v}\right)}^{2}cD}$$

where $\Delta G_{v}$ is the volume free energy change accompanying nucleation, D is the solute diffusivity in the matrix (m^2^/s), $v_{\alpha }$ is the atomic volume during nucleation (m^3^), a is the lattice parameter, and c is the solute concentration in the matrix (mol/L).

#### 2.3.2. Growth and Coarsening

For a spherical precipitate nucleus with radius R and solute concentration $c_{p}\left(t\right)$, its growth process can be analogized to placing such a nucleus in a supersaturated matrix that has an average solute concentration of $c_{m}\left(t\right)$. The spherical precipitate nucleus grows or dissolves through diffusive transport within the matrix, depending on whether the solute concentration $c_{I}\left(R,t\right)$ at the precipitate-matrix interface exceeds $c_{m}\left(t\right)$. Accordingly, the growth or shrinkage rate of the precipitate is expressed as:

(12) $$v\left(R,t\right)=\frac{dR}{dt}=\frac{D}{R}\frac{c_{m}\left(t\right)-c_{I}\left(R,t\right)}{c_{p}\left(t\right)-c_{I}\left(R,t\right)}$$

During the precipitation process of spherical precipitate particles, the precipitates are not uniform in size; the influence of the precipitate size distribution on the interface concentration must be taken into account. Therefore, the equilibrium interface concentration $c_{I}\left(R,t\right)$ is calculated using the nonlinear Gibbs-Thomson equation as follows:

(13) $$c_{I}\left(R,t\right)=c_{me}\left(t\right)\exp\left(\frac{2\gamma \upsilon_{\alpha }}{RK_{B}T}\right)$$

where $c_{me}\left(t\right)$ is the equilibrium concentration in the matrix with a planar interface.

During the entire aging process, the total amount of solute $c^{0}$ partitioned between the matrix and precipitates is conserved. Based on the principle of total solute conservation throughout aging, the following relationship can be derived:

(14) $$c^{0}=\left[1-\varphi \left(t\right)\right]c_{m}\left(t\right)+\varphi \left(t\right)c_{p}(t)$$

Equation (14) can also be expressed as follows: if the solute concentration $c_{p}\left(t\right)$ of the precipitated phase remains unchanged during aging, in practice, during the aging precipitation process of the alloy, the solute concentration $c_{p}\left(t\right)$ of the precipitated phase can be obtained according to the composition of the precipitated phase. Then the average solute concentration $c_{m}\left(t\right)$ in the matrix depends on the volume fraction $\varphi \left(t\right)$ of the precipitated phase. By rearranging Equation (14), obtain:

(15) $$c_{m}\left(t\right)=\frac{c^{0}-\varphi (t)c_{p}(t)}{1-\varphi (t)}$$

According to the precipitate size distribution function $f\left(R,t\right)$ and the radius variation in spherical precipitates, the volume fraction $\varphi \left(t\right)$ of the precipitated phase can be calculated by the following equation:

(16) $$\varphi \left(t\right)=\frac{4\pi }{3}\int_{0}^{\infty }R^{3}f\left(R,t\right)dR$$

While the overall volume fraction remains unchanged, coarsening takes place when larger precipitates grow by consuming smaller ones. Within the population dynamics framework, this phenomenon is driven by the minimization of total interfacial energy. As the solute content in the matrix decreases (i.e., the average solute concentration $c_{m}\left(t\right)$ drops), the driving force for nucleation and growth of the precipitated phase weakens, leading to an increase in the critical nucleation radius $R^{*}$. From Equation (12), precipitate particles with a radius smaller than $R^{*}$ exhibit a negative growth rate and begin to shrink; when the radius of such particles shrinks to zero, they are removed from the size distribution function. Particles with a radius larger than $R^{*}$ show a positive growth rate and continue to grow, with their radii increasing further.

Observing the precipitate radius distribution of the entire system over a long period, smaller precipitate particles disappear, while the size distribution of larger ones continues to grow. Macroscopically, this manifests as a decrease in the number of precipitate particles and an increase in their average radius. Therefore, the coarsening process can be characterized by the total number of precipitate particles $N\left(t\right)$ and their average radius $\bar{R}\left(t\right)$ at time t. The total number $N\left(t\right)$ and average radius $\bar{R}\left(t\right)$ of precipitates at time t can be calculated by the following equations:

(17) $$N\left(t\right)=\int_{0}^{\infty }f\left(R,t\right)dR$$

(18) $$\bar{R}\left(t\right)=\frac{\int_{0}^{\infty }Rf\left(R,t\right)dR}{\int_{0}^{\infty }f\left(R,t\right)dR}$$

#### 2.3.3. Growth Rate Formula with Precipitate Volume Fraction

The growth or dissolution of precipitate particles in the matrix is governed not only by solute diffusion in the matrix, but also by the “competition” among precipitate particles. Marqusee and John found that a “competition” effect exists among precipitate particles, and the competitive growth of these particles is related to the volume fraction [27,28]. Schmidt-Hohagen et al. [29] and Zhao et al. [30] derived the formula for the growth or dissolution rate of precipitate particles under the interparticle “competition” effect:

(19) $$v\left(R,t\right)=\frac{dR}{dt}=\frac{D}{R}\frac{c_{m}\left(t\right)-c_{I}(R,t)}{c_{p}\left(t\right)-c_{I}(R,t)}\left\{1+R{\left[3\varphi \left(t\right)N(t)\bar{R}(t)\right]}^{1/2}\right\}$$

where $\varphi \left(t\right)$ is the volume fraction of the precipitated phase, $N\left(t\right)$ is the number density of precipitate particles, and $\bar{R}\left(t\right)$ is the average radius of precipitate particles.

### 2.4. High-Resolution Model Establishment

#### 2.4.1. Model Discretization

In this paper, the finite volume method is employed to solve the precipitate size distribution function $f\left(R,t\right)$ in the population dynamics model, i.e., the governing Equation (3). The precipitate particle radius R is divided into a large number of uniform intervals. The *i*-th size interval is denoted as $\left[R_{i}-\frac{\Delta R}{2},R_{i}+\frac{\Delta R}{2}\right]$ or $\left[R_{i-\frac{1}{2}},R_{i+\frac{1}{2}}\right]$, where the two endpoints of the interval are the radial vertices of the *i*-th cell, and ΔR is the length of the size interval. We define $f_{i}^{n}$ as the mean value of the precipitate size distribution function $f\left(R,t\right)$ within the *i*-th size interval at time *t*. The value of $f_{i}^{n}$ can then be computed via the following equation:

(20) $$f_{i}^{n}=\frac{1}{∆R}\int_{R_{i-\frac{1}{2}}}^{R_{i+\frac{1}{2}}}f\left(R,t\right)dR$$

The solution of governing Equation (3) can be divided into the following two steps. First, precipitate nuclei formed within a certain time interval are assigned to the appropriate size intervals. For instance, for precipitates formed in the time interval $\left[t_{n},t_{n+1}\right]$, we assume the critical nucleation radius $R^{*}$ lies within the interval $\left[R_{i}-\frac{\Delta R}{2},R_{i}+\frac{\Delta R}{2}\right]$, then $f_{i}^{n}$ satisfies:

(21) $$f_{i}^{n}=f_{i}^{n}+\frac{I∆t}{∆R}$$

For other time intervals, $f_{i}^{n}$ remains unchanged. Combining Equation (3) with Equation (21), it transforms into a variable-coefficient hyperbolic equation:

(22) $$\frac{\partial f(R,t)}{\partial t}+\frac{\partial \left[v\left(R,t\right)f(R,t)\right]}{\partial R}=0$$

In the second step, double integration of Equation (22) is performed over the domain $[R_{i-\frac{1}{2}},R_{i+\frac{1}{2}}]\times \left[t_{n},t_{n+1}\right]$:

(23) $$\int_{R_{i-\frac{1}{2}}}^{R_{i+\frac{1}{2}}}\int_{t_{n}}^{t_{n+1}}\frac{\partial f\left(R,t\right)}{\partial t}dtdR=-\int_{R_{i-\frac{1}{2}}}^{R_{i+\frac{1}{2}}}\int_{t_{n}}^{t_{n+1}}\frac{\partial \left[v(R,t)f\left(R,t\right)\right]}{\partial R}dtdR$$

Integrating and simplifying Equation (23) yields:

(24) $$\int_{R_{i-\frac{1}{2}}}^{R_{i+\frac{1}{2}}}f\left(R,t_{n+1}\right)dR-\int_{R_{i-\frac{1}{2}}}^{R_{i+\frac{1}{2}}}f\left(R,t_{n}\right)dR=-\left[\int_{t_{n}}^{t_{n+1}}v\left(R_{i+\frac{1}{2}},t\right)f\left(R_{i+\frac{1}{2}},t\right)dt-\int_{t_{n}}^{t_{n+1}}v\left(R_{i-\frac{1}{2}},t\right)f\left(R_{i-\frac{1}{2}},t\right)dt\right]$$

given the interfacial fluxes $g\left[f\left(R_{i-\frac{1}{2}},t\right)\right]$ and $g\left[f\left(R_{i+\frac{1}{2}},t\right)\right]$, which represent $v\left(R_{i-\frac{1}{2}},t\right)f\left(R_{i-\frac{1}{2}},t\right)$ and $v\left(R_{i+\frac{1}{2}},t\right)f\left(R_{i+\frac{1}{2}},t\right)$ in Equation (24), respectively. Here, $v\left(R_{i-\frac{1}{2}},t\right)$ and $v\left(R_{i+\frac{1}{2}},t\right)$ are the growth rates at the interfaces, determined by Equation (19). Rearranging Equation (24) gives:

(25) $$\frac{1}{∆R}\int_{R_{i-\frac{1}{2}}}^{R_{i+\frac{1}{2}}}f\left(R,\;t_{n+1}\right)dR=\frac{1}{∆R}\int_{R_{i-\frac{1}{2}}}^{R_{i+\frac{1}{2}}}f\left(R,t_{n}\right)dR-\frac{∆t}{∆R}\left\{\frac{1}{∆t}\int_{t_{n}}^{t_{n+1}}g\left[f\left(R_{i+\frac{1}{2}},t\right)\right]dt-\frac{1}{∆t}\int_{t_{n}}^{t_{n+1}}g\left[f\left(R_{i-\frac{1}{2}},t\right)\right]dt\right\}$$

Combining and simplifying Equation (21) yields:

(26) $$f_{i}^{n+1}=f_{i}^{n}-\frac{∆t}{∆R}\left[F\left(f^{n};i+\frac{1}{2}\right)-F\left(f^{n};i-\frac{1}{2}\right)\right]$$

Equation (26) gives the relationship of the precipitate size distribution function $f\left(R,t\right)$ in adjacent time intervals, where $F\left(f^{n};i-\frac{1}{2}\right)=\frac{1}{∆t}\int_{t_{n}}^{t_{n+1}}g\left[f\left(R_{i-\frac{1}{2}},t\right)\right]dt$ and $F\left(f^{n};i+\frac{1}{2}\right)=\frac{1}{∆t}\int_{t_{n}}^{t_{n+1}}g\left[f\left(R_{i+\frac{1}{2}},t\right)\right]dt$ represent the average fluxes at the left and right interfaces of the i-th interval, respectively. It can be seen from Equations (25) and (26) that the final computational accuracy depends on the interfacial average flux F. Therefore, an appropriate discrete scheme is required to describe the average interfacial fluxes $F\left(f^{n};i-\frac{1}{2}\right)$ and $F\left(f^{n};i+\frac{1}{2}\right)$. To simplify the description, the derivation of subsequent relevant schemes is discussed within the n-th time interval, and the superscript n will be omitted. Based on the derivation of the proposed model, the size interval length ΔR influences the computational accuracy of the size distribution function $f\left(R,t\right)$ to some degree. However, an overly fine size interval will prolong the computational runtime and degrade the overall calculation efficiency. After numerous computational comparisons, a value of ΔR = 0.002 nm is found to be appropriate for the size interval length. The time step used for numerical integration is explicitly determined as $∆t=1\times 10^{-3}$. The high-resolution scheme applied in this work is developed based on the variable-coefficient Lax-Wendroff scheme combined with the Van Leer limiter. The stability of the numerical scheme is strictly evaluated by the Courant-Friedrichs-Lewy criterion. The maximum interfacial growth velocity emerges during the rapid nucleation phase. The CFL number calculated with the maximum growth velocity, the adopted time step and spatial step is less than 1. This result fully satisfies the necessary stability criteria for the applied variable-coefficient Lax-Wendroff scheme. Stable and non-oscillatory numerical solutions can be acquired throughout the entire simulation process, especially during the rapid nucleation stage with the highest growth velocity.

#### 2.4.2. First-Order Upwind Scheme

**Figure 2:**
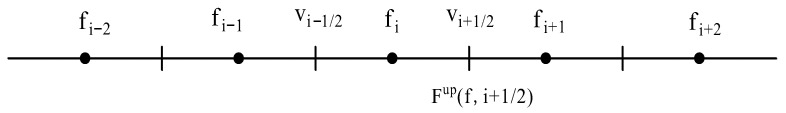
Schematic diagram of the computational grid for the first-order upwind scheme.

Figure 2 shows the schematic of the computational grid based on the first-order upwind scheme, where $F^{up}\left(f;i+\frac{1}{2}\right)$ denotes the average flux at the right interface under the first-order upwind scheme. The growth rate at the interface $v\left(R_{i+\frac{1}{2}},t\right)$ is denoted by $v_{i+\frac{1}{2}}$, which can be calculated by Equation (19). Taking the interface at $F^{up}\left(f;i+\frac{1}{2}\right)$ as an example, two cases must be considered according to the definition of the first-order upwind scheme, leading to the following expressions:

When the interfacial growth rate $v_{i+\frac{1}{2}}>0$, the spherical precipitates grow. According to the first-order upwind scheme, the precipitate size distribution at the right interface is $f\left(R_{i+\frac{1}{2}},t\right)=f_{i}$ and the average interfacial flux is $F^{up}\left(f;i+\frac{1}{2}\right)=v_{i+\frac{1}{2}}f_{i}$.

When the interfacial growth rate $v_{i+\frac{1}{2}}<0$, the spherical precipitates shrink. According to the first-order upwind scheme, the precipitate size distribution at the right interface is $f\left(R_{i+\frac{1}{2}},t\right)=f_{i+1}$, and the average interfacial flux is $F^{up}\left(f;i+\frac{1}{2}\right)=v_{i+\frac{1}{2}}f_{i+1}$.

Expressed in mathematical form:

(27) $$F^{up}\left(f;i+\frac{1}{2}\right)=\left\{\begin{array}{c} v_{i+\frac{1}{2}}f_{i},v_{i+\frac{1}{2}}>0\\ v_{i+\frac{1}{2}}f_{i+1},v_{i+\frac{1}{2}}<0 \end{array}\right.$$

Similarly, the average flux $F^{up}\left(f;i-\frac{1}{2}\right)$ at the left interface under the first-order upwind scheme can be derived.

#### 2.4.3. Lax-Wendroff Scheme

The second-order Lax-Wendroff scheme is derived from Taylor series expansion. For Equation (22), a forward time difference and a central space difference are employed, resulting in a forward-time central-space scheme for this equation. The growth rate $v\left(R,t\right)$ can be regarded as a variable constant a, leading to:

(28) $$\frac{f_{i}^{n+1}-f_{i}^{n}}{∆t}+a\frac{f_{i+1}^{n}-f_{i-1}^{n}}{2∆R}=0$$

Expanding $f_{i}^{n+1}$, $f_{i+1}^{n}$, and $f_{i-1}^{n}$ at node $R_{i}$ and time $t_{n}$ via Taylor series, and omitting higher-order small terms beyond the second order:

(29) $$f_{i}^{n+1}=f_{i}^{n}+{\left(\frac{\partial f}{\partial t}\right)}_{i}^{n}∆t+\frac{1}{2!}{\left(\frac{\partial^{2}f}{\partial t^{2}}\right)}_{i}^{n}∆t^{2}$$

(30) $$f_{i+1}^{n}=f_{i}^{n}+{\left(\frac{\partial f}{\partial R}\right)}_{i}^{n}∆R+\frac{1}{2!}{\left(\frac{\partial^{2}f}{\partial R^{2}}\right)}_{i}^{n}∆R^{2}$$

(31) $$f_{i-1}^{n}=f_{i}^{n}-{\left(\frac{\partial f}{\partial R}\right)}_{i}^{n}∆R+\frac{1}{2!}{\left(\frac{\partial^{2}f}{\partial R^{2}}\right)}_{i}^{n}∆R^{2}$$

Substituting Equations (29)–(31) into Equation (28) yields:

(32) $$\frac{f_{i}^{n+1}-f_{i}^{n}}{∆t}+a\frac{f_{i+1}^{n}-f_{i-1}^{n}}{2∆R}={\left(\frac{\partial f}{\partial t}\right)}_{i}^{n}+a{\left(\frac{\partial f}{\partial R}\right)}_{i}^{n}+\frac{∆t}{2}{\left(\frac{\partial^{2}f}{\partial t^{2}}\right)}_{i}^{n}=0$$

From Equation (22), we have ∂f/∂t=−a∂f/∂R, which leads to the relation $\partial^{2}f/\partial t^{2}=-a\partial^{2}f/\left(\partial t\partial R\right)=a^{2}\partial^{2}f/\partial R^{2}$. Discretizing $\partial^{2}f/\partial R^{2}$ using a three-point central difference gives:

(33) $$\frac{∆t}{2}{\left(\frac{\partial^{2}f}{\partial t^{2}}\right)}_{i}^{n}=\frac{∆ta^{2}}{2}{\left(\frac{\partial^{2}f}{\partial R^{2}}\right)}_{i}^{n}=\frac{∆ta^{2}}{2∆R^{2}}\left(f_{i+1}^{n}-2f_{i}^{n}+f_{i-1}^{n}\right)$$

Substituting Equation (33) into Equation (32) yields:

(34) $$\frac{f_{i}^{n+1}-f_{i}^{n}}{∆t}+a\frac{f_{i+1}^{n}-f_{i-1}^{n}}{2∆R}-\frac{∆ta^{2}}{2∆R^{2}}\left(f_{i+1}^{n}-2f_{i}^{n}+f_{i-1}^{n}\right)=0$$

Rearranging and simplifying Equation (34) according to the form of Equation (26) yields:

(35) $$f_{i}^{n+1}=f_{i}^{n}-\frac{∆t}{∆R}\left\{\left[\frac{a}{2}\left(f_{i+1}^{n}+f_{i}^{n}\right)-\frac{a^{2}}{2}\frac{∆t}{∆R}\left(f_{i+1}^{n}-f_{i}^{n}\right)\right]-\left[\frac{a}{2}\left(f_{i}^{n}+f_{i-1}^{n}\right)-\frac{a^{2}}{2}\frac{∆t}{∆R}\left(f_{i}^{n}-f_{i-1}^{n}\right)\right]\right\}$$

Let $F^{LW}\left(f,i-\frac{1}{2}\right)$ and $F^{LW}\left(f,i+\frac{1}{2}\right)$ denote the average fluxes at the left and right interfaces under the Lax-Wendroff scheme, respectively. By comparing Equation (26) with Equation (35), and replacing the variable coefficient a with the corresponding interfacial growth rates $v_{i-\frac{1}{2}}$ and $v_{i+\frac{1}{2}}$, the average fluxes within the n-th time interval are given by:

(36) $$F^{LW}\left(f,i-\frac{1}{2}\right)=\frac{1}{2}v_{i-\frac{1}{2}}\left(f_{i}+f_{i-1}\right)-\frac{1}{2}{\left(v_{i-\frac{1}{2}}\right)}^{2}\frac{∆t}{∆R}\left(f_{i}-f_{i-1}\right)$$

(37) $$F^{LW}\left(f,i+\frac{1}{2}\right)=\frac{1}{2}v_{i+\frac{1}{2}}\left(f_{i}+f_{i+1}\right)-\frac{1}{2}{\left(v_{i+\frac{1}{2}}\right)}^{2}\frac{∆t}{∆R}\left(f_{i+1}-f_{i}\right)$$

Taking the average flux at the right-hand interface $F^{LW}\left(f,i+\frac{1}{2}\right)$ as an example, this quantity can be split into the right-hand interface average flux of the first-order upwind scheme $F^{up}\left(f,i+\frac{1}{2}\right)$ and a supplementary correction term:

(38) $$F^{LW}\left(f,i+\frac{1}{2}\right)=F^{up}\left(f,i+\frac{1}{2}\right)+\frac{1}{2}\left|\nu_{i+\frac{1}{2}}\right|\left(1-\frac{∆t}{∆R}\left|\nu_{i+\frac{1}{2}}\right|\right)\left(f_{i+1}-f_{i}\right)$$

#### 2.4.4. High-Resolution Scheme

From the mathematical form of Equation (27), it can be seen that the first-order upwind scheme simply sets the interfacial value equal to the value of the upstream node. This allows the first-order upwind scheme to reduce or even avoid the influence caused by large discontinuities in the precipitate size distribution $f_{i}$. Nevertheless, it also introduces numerical diffusion in the computed results, which directly impairs the calculation accuracy and results in relatively large deviations in the calculated ranges.

The Lax-Wendroff scheme obtains the average flux formula at the interface through Taylor expansion, as shown in Equation (38). It can be regarded as the first-order upwind scheme plus a correction term. Owing to this correction term, the Lax-Wendroff scheme yields relatively accurate results when the precipitate size distribution $f_{i}$ is smooth. However, when $f_{i}$ exhibits large discontinuities, the numerical results of the Lax-Wendroff scheme will oscillate under the influence of the precipitate size distribution. Therefore, although the Lax-Wendroff scheme achieves high accuracy in smooth regions, it fails to produce stable solutions in the presence of strong discontinuities.

By comparing Equations (27) and (38), it is observed that the introduced correction term enhances the calculation accuracy, yet it also introduces spurious oscillations into the numerical results. Therefore, an approach of incorporating a flux limiter into the correction term is proposed, which yields a high-resolution scheme with superior computational accuracy:

(39) $$F^{HR}\left(f,\;i+\frac{1}{2}\right)=F^{up}\left(f,i+\frac{1}{2}\right)+\frac{1}{2}\left|\nu_{i+\frac{1}{2}}\right|\left(1-\frac{∆t}{∆R}\left|\nu_{i+\frac{1}{2}}\right|\right)\left(f_{i+1}-f_{i}\right)\Phi_{i+\frac{1}{2}}$$

In the above equations, $F^{HR}\left(f,i-\frac{1}{2}\right)$ and $F^{HR}\left(f,i+\frac{1}{2}\right)$ represent the average fluxes at the left and right interfaces under the high-resolution scheme, respectively. The expression for the limiter $\Phi_{i+\frac{1}{2}}$ is given as follows:

(40) $$\Phi_{i+\frac{1}{2}}=\Phi \left(\theta_{i+\frac{1}{2}}\right)$$

Here, θ denotes the ratio of successive gradients, which is primarily employed to describe the “smoothness” of the numerical data. For example, the ratio of consecutive gradients $\theta_{i+\frac{1}{2}}$ at the right interface of the i-th interval is given by:

(41) $$\theta_{i+\frac{1}{2}}=\frac{f_{I+1}{-f}_{I}}{f_{i+1}{-f}_{i}}$$

In the above Equation (40), the subscript I is defined as:

(42) $$I=\left\{\begin{array}{c} i-1,v_{i+\frac{1}{2}}>0\\ i+1,v_{i+\frac{1}{2}}\leq 0 \end{array}\right.$$

From Equations (39)–(42), it can be seen that for the high-resolution scheme, the computational accuracy and stability of the results mainly depend on the formulation of the limiter $\Phi_{i+\frac{1}{2}}$. Several limiter expressions have been proposed in the study of fluid dynamics. Sweby conducted numerical studies based on these limiters and found that the limiter proposed by Van Leer performs well in solving shock wave problems in fluid dynamics [31]. When solving the population dynamics model, the discontinuous jump problem of the precipitate size distribution $f_{i}$ is analogous to the shock wave problem in fluid dynamics. Therefore, the limiter proposed by Van Leer is also applicable to the above problem. The formulation of the Van Leer limiter is given by:

(43) $$\Phi \left(\theta \right)=\frac{\theta +\left|\theta \right|}{1+\left|\theta \right|}$$

With the Van Leer limiter introduced, a high-resolution scheme with second-order accuracy is obtained. In fact, according to the formulation of Equation (39), this high-resolution scheme adopts the second-order Lax-Wendroff scheme in smooth data regions and the first-order upwind scheme in non-smooth regions. The Van Leer limiter effectively combines the two schemes, reducing the numerical influence caused by data discontinuities.

## 3. Results and Discussion

### 3.1. Thermodynamic Calculation of Al-Sc Alloys

The Al_3_Sc precipitated phase can markedly enhance the recrystallization resistance, mechanical strength and high-temperature creep performance of Al-Sc alloys. Therefore, it is of great research value to clarify its precipitation behavior during aging. In this study, based on a high-resolution population dynamics model, the effects of interfacial energy evolution and precipitate volume fraction were simultaneously taken into account. Simulations were carried out for the isothermal aging process of two alloys, Al-0.2 wt. %Sc and Al-0.3 wt. %Sc, at 350 °C, to investigate the precipitation behavior of the Al_3_Sc phase.

In this study, the thermodynamic data for the Al-Sc alloy system reported by Murray [32] were adopted to calculate the volume free energy $\Delta G_{v}$ and the equilibrium concentration $c_{me}$. The functional relationship of the diffusion coefficient *D* of Sc in the Al matrix was derived from three sets of data: the calculated results of Watanabe [33] based on the LSW theory, the data obtained by Fujikawa [34] using the tracer diffusion technique, and the coarsening experimental data of particle size measured by Marquis and Seidman [35] via TEM:

(44) $$D=\left(7.2\pm 6.0\right)\times 10^{-4}\times exp\left(\frac{-176000\pm 9000}{R_{g}T}\right)$$

According to Equation (44), the diffusion coefficient of Sc in the Al matrix at the isothermal aging temperature of 350 °C (623.15 K) is calculated to be $D=1.27\times 10^{-18}\;m^{2}/s$.

Consistent with the treatment in Robson’s study [24], the present work also considers the continuous interfacial energy evolution between the nucleation and coarsening stages to prevent any unphysical numerical shocks. Combined with the findings of Hyland [36] and comparisons between multiple sets of simulation and experimental data, the interfacial energy in the initial nucleation stage is determined to be 118 mJ/m^2^. For the coarsening stage, the interfacial energy adopts the first-principles calculation result of Asta [37], with a value of 200 mJ/m^2^. To smoothly bridge these two regimes computationally, a continuous linear interpolation function dependent on the precipitate radius was utilized. Specifically, as the particle radius grows to 5 nm, the interfacial energy increases linearly from 118 mJ/m^2^ to 200 mJ/m^2^, and it remains constant at 200 mJ/m^2^ for radii larger than 5 nm. This continuous transition ensures computational stability and confirms that the observed plateau in the average radius evolution is a genuine physical phenomenon rather than a numerical artifact. The simulation results are compared with the experimental data of Novotyn [38] and the simulation results based on the LSW theory, and the accuracy of the model is verified by goodness-of-fit analysis.

### 3.2. Simulation Results of Al-0.2 wt.% Sc Alloy

**Figure 3:**
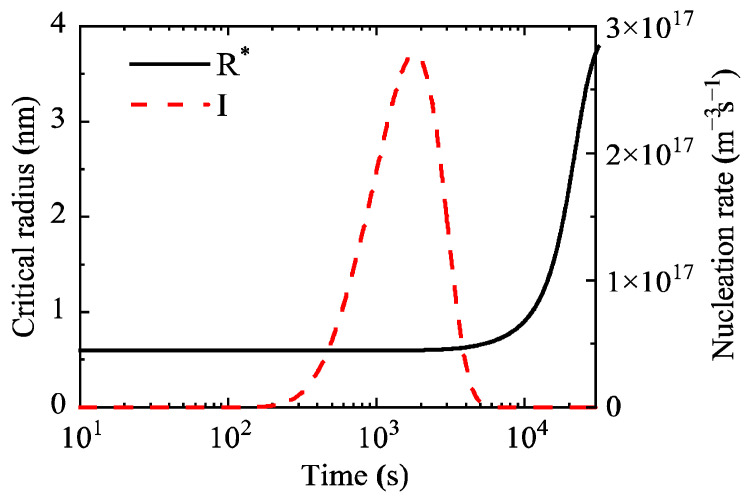
Critical nucleation radius (R*) and nucleation rate (I) of precipitates during aging at 350 °C in the Al-0.2 wt.% Sc alloy.

Simulation of the precipitation behavior of Al_3_Sc in the Al-0.2 wt.% Sc alloy was carried out, and the temporal evolution laws of characteristic parameters such as the average precipitate radius and nucleation rate during aging were obtained. Figure 3 shows the temporal evolution of the critical nucleation radius and nucleation rate of precipitates in the Al-0.2 wt.% Sc alloy during aging. The results reveal that the nucleation process lasts approximately 5800 s, and the peak nucleation rate reaches 2.76 × 10^17^ m^−3^s^−1^.

**Figure 4:**
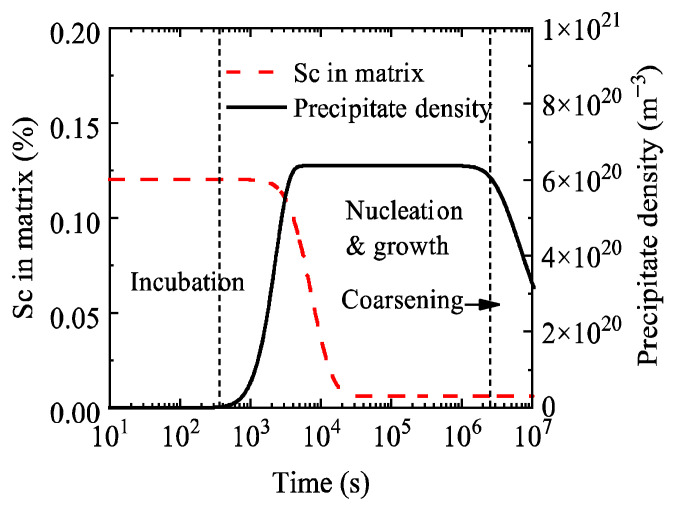
Temporal evolution of precipitate number density and Sc content in the matrix during aging of the Al-0.2 wt.% Sc alloy.

Figure 4 shows the evolution curves of the number density of Al_3_Sc precipitates and the Sc content in the matrix during aging of the Al-0.2 wt.% Sc alloy. The precipitate number density starts to increase at approximately 200 s and reaches a peak at around 6000 s, which is close to the duration of the nucleation stage in Figure 3. It then remains stable until it begins to decrease at about 3 × 10^6^ s, consistent with the onset time of the alloy coarsening stage.

In Figure 4, the aging process is divided into three stages: incubation, nucleation-growth, and coarsening. During the nucleation-growth stage, the Sc content in the matrix decreases from 0.12% to 0.01%, and the solute is mainly consumed for the formation and growth of Al_3_Sc precipitates. After entering the coarsening stage, the Sc content in the matrix reaches the equilibrium concentration, and the precipitates enter a growth stage where large particles grow at the expense of small particle dissolution, which leads to a decrease in the total particle number density within the system.

**Figure 5:**
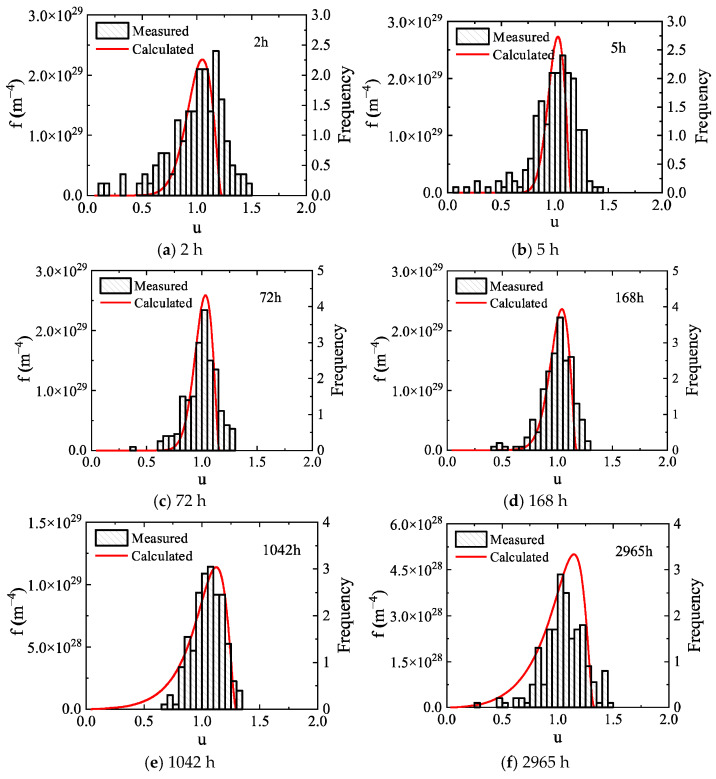
Comparison of calculated and experimentally measured normalized size distributions of precipitates during aging in the Al-0.2 wt.% Sc alloy. Here, f represents the precipitate size distribution function, and u denotes the dimensionless normalized particle radius.

**Figure 6:**
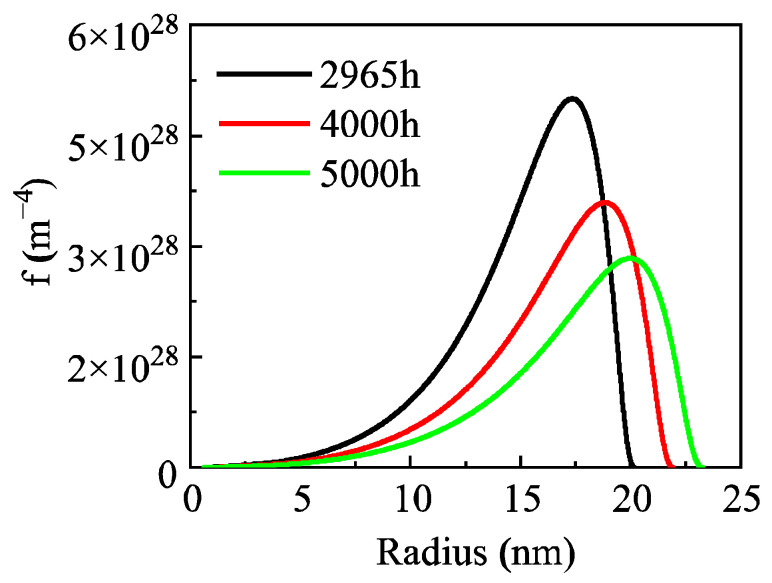
Prediction of precipitate size distribution during aging in the Al-0.2 wt.% Sc alloy.

Novotyn and Ardell’s research shows that the normalized size distribution of precipitates in the Al-0.2 wt.% Sc alloy narrows in the early aging stage and gradually broadens with prolonged aging time in the later stage, with their experimental data presented as histograms in Figure 5. The normalized size distribution curves calculated by the high-resolution model in this study agree well with the experimental data. Both show a narrowing trend during the aging period of 2–168 h. In the aging stage of 168–2965 h, the size distribution of the Al_3_Sc phase gradually broadens with increasing time. In addition, the model also predicts the size distribution after aging for 4000 h and 5000 h (Figure 6), indicating that the size distribution of the Al_3_Sc phase still maintains a broadening trend under longer aging times.

### 3.3. Simulation Results of Al-0.3 wt.% Sc Alloy

**Figure 7:**
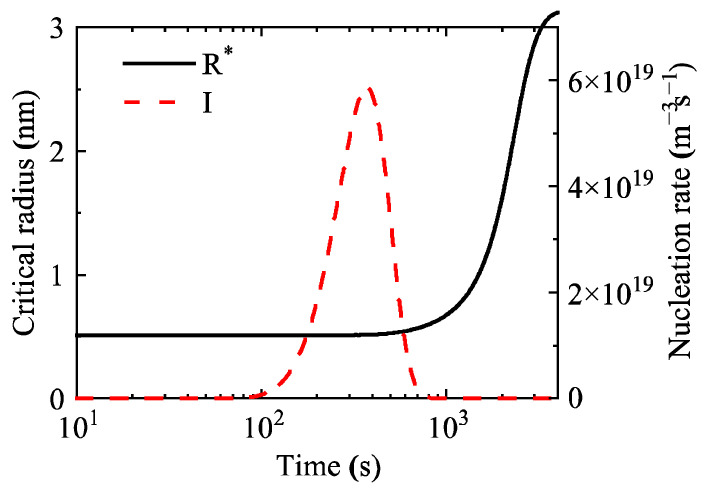
Critical nucleation radius (R*) and nucleation rate (I) of precipitates during aging at 350 °C in the Al-0.3 wt.% Sc alloy.

For the simulation study on the isothermal aging process of the Al-0.3 wt.% Sc alloy at 350 °C, the temporal variations in characteristic parameters such as the average precipitate radius and nucleation rate were also calculated. Figure 7 shows the evolution laws of the critical nucleation radius and nucleation rate of precipitates during aging of this alloy. The results indicate that the nucleation process lasts only about 800 s, and the critical nucleation radius increases gradually after nucleation is completed. Compared with the Al-0.2 wt.% Sc alloy, the nucleation duration of this alloy is significantly shortened, while the peak nucleation rate is higher.

**Figure 8:**
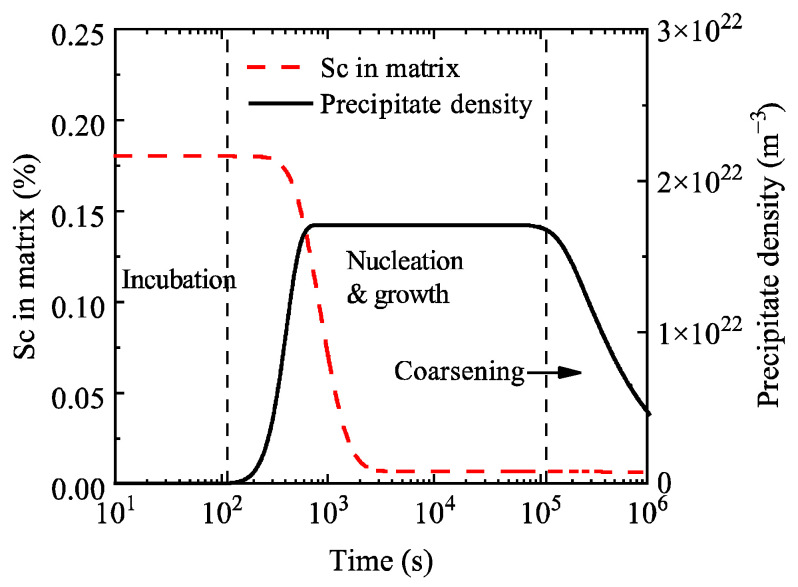
Temporal evolution of precipitate number density and Sc content in the matrix during aging of the Al-0.3 wt.% Sc alloy.

Figure 8 shows the evolution curves of precipitate number density and Sc content in the matrix during aging of the Al-0.3 wt.% Sc alloy. The precipitate number density starts to increase at approximately 100 s and reaches a peak at around 900 s, consistent with the duration of the nucleation stage. It then remains stable until it begins to decrease at about 1 × 10^5^ s, corresponding to the onset time of the coarsening stage.

With reference to the classification adopted for the Al-0.2 wt.% Sc alloy, the aging process of the Al-0.3 wt.% Sc alloy is divided into three stages—incubation, nucleation-growth, and coarsening—as labeled in Figure 8. During the nucleation-growth stage, the Sc content in the matrix decreases from 0.18% to 0.01%. In the coarsening stage, the Sc content in the matrix reaches the equilibrium concentration, which is basically consistent with that of the Al-0.2 wt.% Sc alloy owing to the identical aging temperature.

By comparing Figure 4 and Figure 8, it can be seen that an increase in Sc content significantly shortens the duration of the nucleation-growth stage and substantially advances the onset time of the coarsening stage. The main reason is that the increased Sc content raises both the precipitate volume fraction and particle number density. In the coarsening stage, the growth of large particles is achieved through the dissolution of small particles, which ultimately accelerates the entire aging precipitation process.

**Figure 9:**
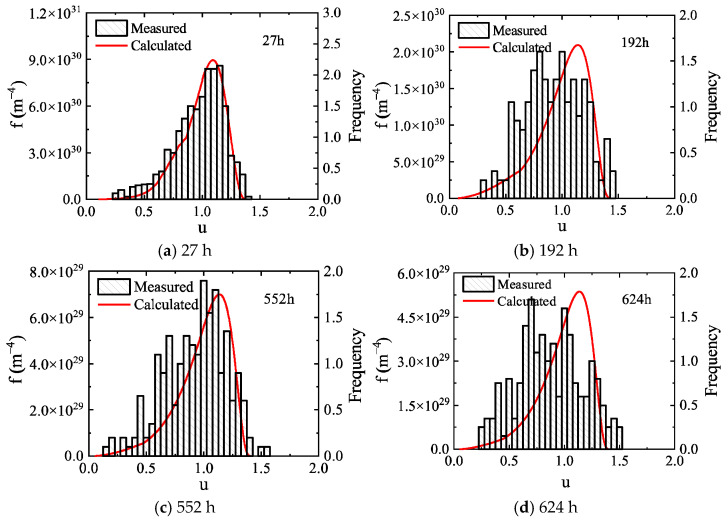

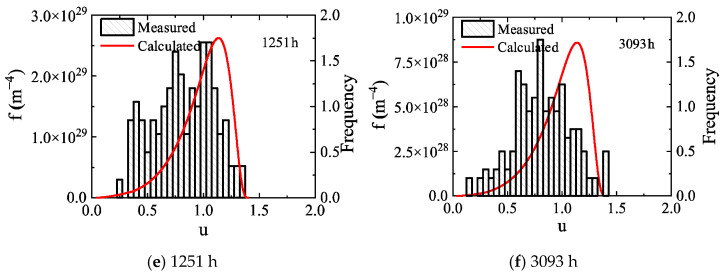
Comparison of calculated and experimentally measured normalized size distributions of precipitates during aging in the Al-0.3 wt.% Sc alloy. Here, f represents the precipitate size distribution function, and u denotes the dimensionless normalized particle radius.

The normalized size distribution of precipitates in the Al-0.3 wt.% Sc alloy measured experimentally by Novotyn is shown as histograms in Figure 9. Different from the Al-0.2 wt.% Sc alloy, the size distribution of the Al-0.3 wt.% Sc alloy broadens continuously with prolonged aging. The size distribution curves predicted by the high-resolution model employed in this work are in good agreement with the experimental measurements during the early stage of aging. In the later aging stage (after approximately 624 h), a slight deviation appears in the normalized size corresponding to the distribution peak, but the distribution widths of the two remain relatively close.

**Figure 10:**
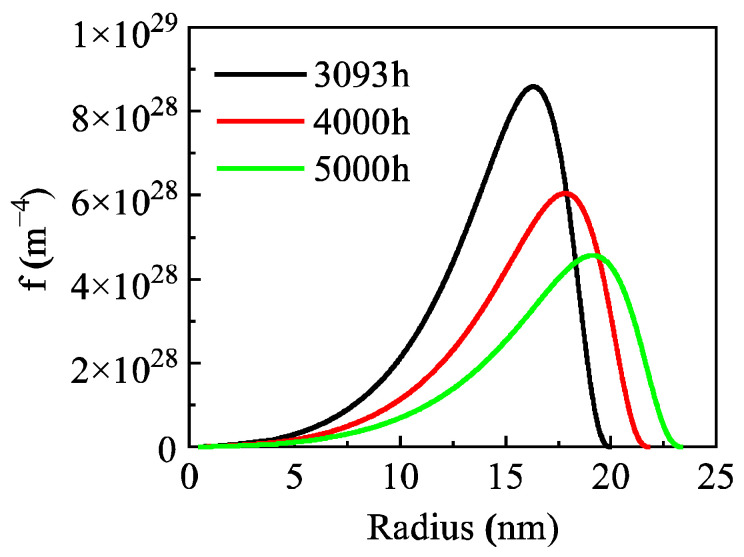
Prediction of precipitate size distribution during aging in the Al-0.3 wt.% Sc alloy.

The main reason for the deviation is that both the present model and the traditional LSW theory are based on the assumption of spherical precipitate particles, whereas the Al_3_Sc particles in the Al-0.3 wt.% Sc alloy transform from spherical to cubic morphology with increasing aging time, which in turn affects the size distribution [38]. To rigorously capture this morphological evolution, a size-dependent geometric shape factor that continuously corrects the surface-area-to-volume ratio must be integrated into the governing KWN framework. Given the algorithmic complexities inherent in coupling this transition, such an extension is reserved for future investigations. The model also predicts the size distribution at longer aging times, as shown in Figure 10. With extended aging time, the precipitate size distribution continues to broaden and the distribution peak decreases gradually.

### 3.4. Comparative Analysis of Precipitation Behavior Under Different Theoretical Models

**Figure 11:**
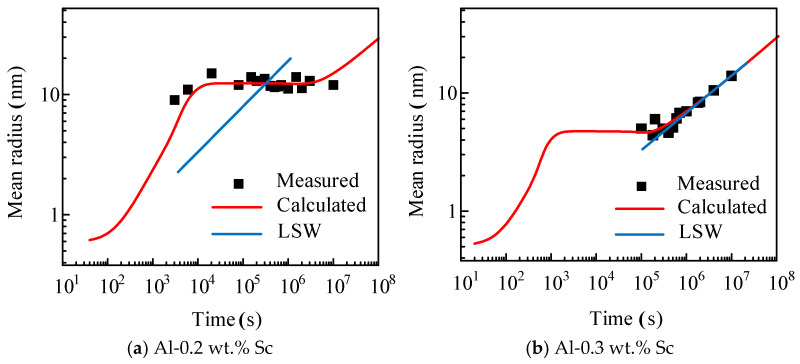
Comparison of average precipitate radii of two alloys under isothermal aging at 350 °C: results of high-resolution algorithm, LSW theory and experimental data.

Figure 11 compares the calculated results from the high-resolution population dynamics model and the conventional LSW theory with experimental data of the average precipitate radius for the two alloys under isothermal aging at 350 °C. As shown in Figure 11a, for the Al-0.2 wt.% Sc alloy, the simulation curve of the high-resolution model clearly reproduces the evolution law of the average precipitate radius. It enters a plateau after approximately 10^4^ s and formally transitions to the coarsening stage at around 3 × 10^6^ s, exhibiting typical LSW coarsening characteristics after the plateau. As shown in Figure 11b, for the Al-0.3 wt.% Sc alloy, the curve of the average Al_3_Sc precipitate radius calculated by the high-resolution model not only reproduces the LSW coarsening characteristic of the linear growth of the average radius with aging time in the experimental data, but also accurately captures the evolution feature that the average radius enters a plateau at about 10^3^ s and shifts to the coarsening stage at around 10^5^ s. The duration of the plateau is significantly shorter than that of the Al-0.2 wt.% Sc alloy. It should be clarified that the LSW theory is only applicable to the precipitate coarsening stage. The prediction deviation of the Al-0.2 wt.% Sc alloy using the LSW theory does not mean that the alloy exhibits no LSW coarsening behavior. Instead, the alloy possesses higher coarsening resistance and remains in the nucleation-growth plateau for most of the experimental observation period without entering the applicable range of the LSW theory, which is the core reason why the LSW theory fails to accurately predict its evolution law.

The high-resolution population dynamics model established in this study can accurately reproduce the experimental laws of both alloys. For the Al-0.2 wt.% Sc alloy, the experimentally measured average radius is concentrated in the plateau region of the nucleation-growth stage without an obvious growth trend. The model not only reproduces the transition of the average radius from rising to leveling off in the early aging stage, but also accurately predicts the typical LSW coarsening characteristics after the plateau. For the Al-0.3 wt.% Sc alloy, the model agrees well with the linear growth law of the average radius in the experiments and reasonably explains the early plateau phenomenon that cannot be accounted for by the LSW theory. The calculated results of the average precipitate radius and normalized size distribution for both alloys are in high agreement with the experimental data, proving that the high-resolution population dynamics model is reliable in accuracy and provides a more accurate description of the aging precipitation behavior of the alloys.

Further comparison of the size distribution results demonstrates that the predicted values for the Al-0.2 wt.% Sc alloy exhibit excellent consistency with the experimental data across the entire aging duration from 2 h to 2965 h. This is because the aging time required for the precipitate particles in this alloy to reach the critical size for morphological transformation is extremely long, and no transformation from spherical to cubic morphology occurs within the experimental observation range. In contrast, the Al-0.3 wt.% Sc alloy shows good agreement only in the early aging stage, and the deviation gradually increases when the aging time exceeds 624 h. The main reason is that the precipitates undergo a morphological transformation from spherical to cubic, which goes beyond the applicable range of the spherical particle assumption in the model. The above results indicate that the simulation results of the high-resolution population dynamics model established in this study are highly reliable within the aging range before morphological transformation of the precipitate particles.

## 4. Conclusions

Aiming at solving the key problems that the traditional Lifshitz-Slyozov-Wagner (LSW) theory is only applicable to the precipitate coarsening stage and cannot fully characterize the precipitation kinetics during the entire aging process of Al-Sc alloys and that the conventional Kampmann-Wagner-Numerical (KWN) model suffers from numerical diffusion and is difficult to correct the calculation deviation in the nucleation stage via high-order algorithms, this study established a high-resolution population dynamics model based on the modified KWN model. The model simultaneously incorporates the effects of interfacial energy evolution and precipitate volume fraction, and systematically simulates the precipitation behavior of nanoscale Al_3_Sc phases in Al-0.2 wt.% Sc and Al-0.3 wt.% Sc alloys during isothermal aging at 350 °C. Key kinetic parameters such as average precipitate radius, normalized size distribution, and nucleation rate were calculated, and the simulation results were compared and verified with experimental data and LSW theoretical calculations. The main conclusions are as follows:

(1) The high-resolution population dynamics model constructed in this study achieves a solution effect of second-order computational accuracy in smooth regions and no spurious oscillations in discontinuous regions by introducing the Van Leer limiter. It effectively solves the numerical diffusion problem existing in the traditional first-order upwind scheme and fully reproduces the precipitation kinetics of the three consecutive stages during aging of Al-Sc alloys: incubation, nucleation-growth, and coarsening. Both the average radius and the normalized size distribution of Al_3_Sc precipitates obtained from the present model show excellent agreement with the available experimental data reported in the literature. The model can accurately capture the plateau characteristics of the average precipitate radius evolution and the evolution rule of “first narrowing, then broadening” in the size distribution during aging. Compared with the traditional LSW theory, which can only describe the behavior in the coarsening stage, the present model possesses higher prediction accuracy and a wider application range.
(2) Sc content has a significant regulatory effect on the aging precipitation kinetics of Al-Sc alloys. An increase in Sc content raises the equilibrium volume fraction and number density of precipitates, accelerates the coarsening process of large particles at the expense of small-particle dissolution during the coarsening stage, significantly shortens the duration of the nucleation and growth stage of the alloy, and substantially advances the onset time of the coarsening stage. Among them, the nucleation process of the Al-0.3 wt.% Sc alloy lasts only about 800 s, which is considerably shorter than the 5800 s of the Al-0.2 wt.% Sc alloy. Meanwhile, the onset time of the coarsening stage is advanced by approximately one order of magnitude relative to that of the Al-0.2 wt.% Sc alloy.

The high-resolution population dynamics model established in this study accurately describes the evolution behavior of precipitates during the full aging cycle of Al-0.2 wt.% Sc and Al-0.3 wt.% Sc alloys. It can not only provide reliable theoretical support for the optimal design of aging heat treatment processes for Al-Sc alloys, but also offers an efficient and accurate implementation method for the numerical simulation of precipitation kinetics in other binary alloy systems. Future research can extend the present model to the simulation of precipitation kinetics for non-spherical precipitates, and establish a quantitative correlation between the microstructural characteristics of precipitates and the macroscopic mechanical properties of alloys, so as to further expand the engineering application value of the model.

## Data Availability

The original contributions presented in this study are included in the article. Further inquiries can be directed to the corresponding authors.
